# Causal Attribution and Illness Perception: A Cross-Sectional Study in Mexican Patients with Psychosis

**DOI:** 10.1155/2014/969867

**Published:** 2014-12-01

**Authors:** Lizzette Gómez-de-Regil

**Affiliations:** Hospital Regional de Alta Especialidad de la Península de Yucatán (HRAEPY), Calle 7, No. 433, por 20 y 22, Fraccionamiento Altabrisa, 97130 Merida, YUC, Mexico

## Abstract

Health psychology researchers have begun to focus greater attention on people's beliefs about health/illness since these beliefs can clearly affect behavior. This cross-sectional study aimed at (1) identifying the most common factors psychotic patients attribute their illness to and (2) assessing the association between causal attribution and illness perception (cognitive, emotional, and comprehensibility dimensions). Sixty-two patients (56.5% females) who had been treated for psychosis at a public psychiatric hospital in Mexico answered the Angermeyer and Klusmann Illness Attribution Scale and the Brief Illness Perception Questionnaire. Results showed that most patients attributed psychosis onset to social factors and that attribution to their personality might have an overwhelmingly negative effect on their lives. Acknowledging psychotic patient attributional beliefs and considering them in clinical practice could improve treatment efficacy and overall recovery success. This is particularly important in psychosis, since symptoms are often severe and/or persistent and require long-term treatment.

## 1. Introduction

Health care needs to go beyond mere treatment of illness symptoms and consider the patient as a whole. Clinicians must involve patients not only during examination to generate a diagnosis or prognosis but also when considering treatment options. This process involves the patient as a coresponsible party and can produce a voluntarily commitment from him/her to fulfill the treatment and, if necessary, change beliefs and behaviors that may affect outcome. Recent studies suggest that patient self-determination and self-control need to be promoted to improve mental illness treatment adherence and recovery. This can be accomplished through shared decision making, allowing patients to feel understood and valued, and helping them develop a sense of independence and efficacy [1–3].

Research has increasingly focused attention on people's beliefs about health/illness since these can clearly affect behavior. A patient's perception of illness may influence the probability of seeking treatment [4, 5] and treatment adherence [6–8], and it can also affect disability and recovery time [9, 10], use of medical services [11, 12], quality of life [13–16], self-esteem, anxiety, and depression [13, 17, 18]. As a result, exploring patients' perceptions of their illness is becoming an important aspect of clinical practice.

After symptom onset, patients elaborate individual cognitive and emotional representations of their disorder, integrating their medical knowledge (accurate or not) with previous known experiences of themselves, relatives, or acquaintances with similar symptoms or diagnoses. These representations guide patients' actions aimed at controlling their symptoms/illness and the strategies they apply to confront their emotional impact [19]. The first dimension of illness perception to be explored was cognitive representation, which involves beliefs about the cause of the illness, the expected physical consequences, the illness's emotional or functional effects on life, the extent to which a patient believes he/she can recover from it, if recovery will occur with or without treatment, how the illness and its symptoms are identified and named, and ideas about how long it will last [19, 20]. Recent studies have included the emotional dimension, which focuses on negative reactions such as fear, anger, and distress, and the understanding dimension, that is, the perception of to what extent patients feel they comprehend the dynamics of their illness [21].

Illness causal attribution has been a major research focus in both patients [5, 22, 23] and their relatives [24–26]. In their attempt to face their illness, individuals integrate the information provided by the clinician with their own to interpret why the illness has occurred. Causal attribution of illness influences the type of treatment patients seek and the actions they take to control symptoms. Beliefs about an illness's etiology may also affect patient emotional response, particularly in illness in which the etiology is still unclear [27]. Psychosis has been attributed to social, personality, family, biological, and even esoteric factors [23, 24]. These causal beliefs can positively or negatively influence patient expectations about prognosis and to what extent they take an active role in recovery.

The present study aimed at identifying the most common factors psychotic patients attribute their illness to and at assessing the association between causal attribution and the three dimensions of illness perception (cognitive, emotional, and comprehensibility). The relationship between etiological beliefs and illness perception has not yet been addressed in the literature. Causal attribution and illness perception in patients with psychosis have been studied, although no data from Mexico or other Latin American countries has yet been published. Understanding patients' beliefs about their illness is particularly important in psychosis since symptoms are often severe and/or persistent and require long-term treatment. Addressing these issues in new samples like the one presented here will allow identification of similarities and differences in the beliefs of psychotic patients from different cultures and supply original data on the studied population. A broader awareness in clinical practice of psychotic patient beliefs regarding their illness will contribute to ongoing efforts to improve treatment efficacy.

## 2. Methods

### 2.1. Participants

Participants were patients who had received mental health care at the adult service of the Yucatan Psychiatric Hospital in Merida, Mexico. All were residents of the city of Merida. The protocol for this cross-sectional study was approved and authorized by the Yucatan Psychiatric Hospital Committee and performed over a 12-month period. The protocol adhered to international [28] and national [29] ethical standards for studies with minimal risk. When invited to participate, patients were guaranteed confidentiality and emphasized that their decision to accept or to decline would not condition the attention provided by the hospital or its quality and they were free to withdraw from the study at any time. Informed consents were signed by participants and one of their relatives as witness, and a copy was given to them with the researcher contact information.

First, clinical files were reviewed to identify patients meeting the following criteria: (i) 16–45 years of age at onset and (ii) a primary current DSM-IV-TR [30] diagnosis of schizophrenia or other schizophrenia-spectrum psychotic disorders. Exclusion criteria were (i) DSM-IV-TR diagnosis of affective, organic, or toxic type psychosis [30]; (ii) presence of an evident intellectual disorder; and (iii) inadequate contact information.

**Figure 1:**
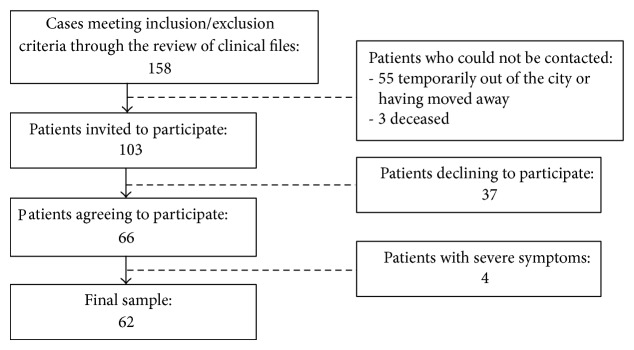
Flow of participant recruitment.

From 158 potential cases, final sample included 62 participants (56.5% females) who agreed to be interviewed with no economic compensation involved (Figure 1). Thirty-four (55%) participants had elementary and/or partial middle school education levels (up to 9th grade) and the remaining 28 (45%) had partial/complete middle or high school education levels. None of the participants was hospitalized at the time of the assessment. In terms of current DSM-IV-TR diagnoses, 42 patients had schizophrenia (14 paranoid, 3 disorganized, and 25 residual) and 20 patients had other types of schizophrenia-spectrum psychoses (8 schizoaffective, 7 delusional, 2 schizophreniform, 2 brief, and 1 not otherwise specified). Mean illness course was 6.8 years (SD = 1.9, range 3.8–11.2). Current mean age was 35.8 years (SD = 9.9). Mean age at onset was 29.0 years (SD = 9.8). No significant sex differences were found in relation to diagnosis, illness course time, current age, and age at onset.

### 2.2. Instruments

Illness perception was measured with the Spanish version of the Brief Illness Perception Questionnaire (Brief-IPQ) [21, 31]. This nine-item self-report scale assesses three dimensions of illness perception: cognitive representation (beliefs about illness severity, consequences, and duration, personal control over illness, and treatment usefulness), emotional representation (negative emotions regarding illness), and comprehensibility (understanding of the disorder). Higher scores of cognitive and emotional representations indicate an unfavorable perception of illness, whereas higher scores of comprehensibility reflect satisfactory understanding of the disorder. Although this scale is not exclusive for people with a mental illness, it has been satisfactorily applied to patients with psychosis [32]. Adequate psychometric properties have been reported for this instrument in its original version [21] and adaptations to other languages, including Spanish [31, 33–35]. Item nine of the Brief-IPQ is an open question requesting participants to mention three factors that they believe caused their illness. To more broadly assess causal attribution, this item was replaced by the Angermeyer and Klusmann [23] Scale listing 30 possible causes in five categories (Biology, Personality, Family, Society, and Esoteric), an instrument with satisfactory psychometric qualities [36]. Both scales were presented in a four-point Likert format; items were read aloud by the researcher requesting participants to respond with the visual support of four drawn squares, from the smallest (“not a cause”/“totally disagree”) to the largest (“very likely a cause”/“totally agree”). This dynamic for the interview was followed due to the fact that from the initial outlook of clinical files a predominant low level of education was evident, which could have made it harder for participants to respond. This alternative procedure intended not only to improve the reliability of data but also to make participants feel comfortable with the interview.

### 2.3. Statistical Analysis

Descriptive statistics were obtained for causal attribution and illness perception. Pearson two-tailed correlations were used to analyze their association. All tests were run with the SPSS 15.0 software.

## 3. Results

“Stressful life events” was the factor most frequently attributed as “(very) likely” the cause of illness, followed by “Disturbance of brain biochemistry,” “Constant strain in school/job,” “Avoidance of everyday life problems,” and “Failure in life.” This pattern was confirmed by item total scores. Two of these four factors were attributed to Society and the other two to Personality. Total score by category exhibited a preference for Society factors, followed by Personality, Family, Biology, and Esoteric (Table 1). No significant differences were found when comparing groups by sex, educational level, or diagnosis; current age and age at onset showed no significant correlations with any category score. The average numbers of items attributed as “(very) likely” are as follows: overall, 8.2 (SD = 5.4); Biology, 1.4 (SD = 1.4); Personality, 2.0 (SD = 1.6); Family, 1.7 (SD = 1.7); Society, 2.3 (SD = 1.6); and Esoteric, 0.8 (SD = 1.2).

Illness perceptions were favorable for the three dimensions: cognitive (mean = 2.13, SD = 0.58), emotional (mean = 2.50, SD = 1.05), and understanding (mean = 2.85, SD = 1.14). As with attribution, sex, educational level, diagnosis, current age, and age at onset were not related to illness perception scores. All three illness perception dimensions (cognitive, emotional, and understanding) were significantly (*P* ≤ .05) related to illness attribution to the Personality and Family categories. Moderate correlation values (*r* ≥ .30) were observed only in relation to the emotional dimension. A small but significant correlation was identified between Biology and the emotional dimension (Table 2).

Analysis of the individual illness perception items revealed some small (*r* < .30) but significant (*P* ≤ .05) correlations with the overall Biology, Personality, Family, and Society attribution scores. Moderate (*r* ≥ .30) and significant (*P* ≤ .05) correlations were found between attribution to Personality and the subjective perception of illness as negatively affecting patient emotional status and life in general (Table 3).

## 4. Discussion

Illness perceptions, beliefs, and attributions have increasingly become the focus of research for health psychology, psychiatry, and related disciplines. Although some research related to these topics has been performed in Mexican populations [37, 38], data regarding psychiatric disorders or alike clinical conditions are still scarce [37, 39, 40].

The first study aim was to identify the causes of psychosis most commonly endorsed by patients. The causal attributions documented in the present study were largely social factors, which agrees with previous research showing a preference for attribution to social factors as the cause of psychosis [22, 23], followed by Personality, Biology, Family, and Esoteric factors [23]. The tendency to identify social factors as the etiological agents behind psychosis is independent of age, sex, and educational level [23, 41] and was also replicated. A patient preference for social factors as the cause of psychosis has also been identified in German [22] and Greek [22, 41] samples, suggesting that attribution to social factors may be independent of patient cultural origin. This requires a better understanding of the learning mechanisms driving patients to create a model in which social factors are the causal agents behind their psychosis and of whether or not this pattern is specific to mental disorders. Moreover, these results might well fit into the study frame of the social determinants of health (or illness) promoted by the World Health Organization [42], providing evidence of social factors being acknowledged by patients with psychosis as a major influence triggering their illness. Should this attribution of psychosis to social factors be replicated in the general population, it will serve not only to design suitable campaigns promoting mental health awareness but also to identify possible social conditions in the community triggering psychosis that could be modified.

The second study aim was to assess the association between causal attribution of psychosis and the three illness perception dimensions (cognitive, emotional, and understanding). Attribution to Biology was related to the emotional dimension (“I am extremely concerned about my illness”). Attribution to Personality and/or Family was associated with higher scores in the cognitive dimension (“My illness affects my life”) and emotional dimension (“I am extremely concerned about my illness” and “My illness extremely affects me emotionally”) and lower scores in the understanding dimension (“I very clearly understand my illness”). This suggests that attribution of psychosis to factors proximal to the patient may be associated with feelings of being overwhelmed by the illness along with poor disorder comprehension. These issues deserve more qualitative exploration to distinguish any possible psychological interpretations of illness underlying the patterns. Worth highlighting is the association of the patients' attribution of illness to personality with their perception of the illness negatively affecting their life and emotional status. This is congruent with research showing that, in patients with a chronic illness, an internal locus of control is associated with a higher degree of psychological distress [43, 44]; when patients see themselves as responsible for illness onset, they may experience feelings of guilt. However, in the present data, causal attribution to personality (i.e., internal) factors exhibited no significant association with illness control (personal or treatment). This suggests that causal attribution of psychosis to internal factors is associated with a greater perception of negative illness consequences but not to control of illness evolution. Given the above, psychotic patients with a high degree of internal attribution could benefit from stimulation of their perception of control of the illness and of treatment efficacy aimed at reducing their psychological discomfort. That said, patients suffering serious mental illness report a sense of personal futility and powerlessness in terms of improving their health, even though they express interest in learning about health promotion. Self-efficacy needs to be promoted in these patients to improve their well-being through development of their sense of independence, value, and self-control [3, 45].

The present study design contains some primary limitations in terms of controlling variables that could influence illness attribution and perception, including previous exposure to formal or informal information about mental illness, personality traits, and severity of psychotic episode(s), among others. Regarding sample, a significant number of patients could not be contacted, diminishing sample potential size and population representativeness. Moreover, it included only inhabitants from the city, leaving open the question whether these patterns of ideas could be replicated in patient samples from proximate rural areas where community has a more significant influence on the individual but also where esoteric beliefs are more openly acknowledged. The interview with scales in a Liker format, although favorable for analyses and interpretation, made it clear that, in samples with low educational level and/or severe pathologies, adaptation is required. Furthermore, though consistent with previous studies in samples from Mexico [46], sample mean age at the onset of psychosis (29 years) might seem atypical. Whether psychosis actually has a later onset in Mexican population and/or patients have a longer period of untreated psychosis before seeking treatment cannot be concluded from the available data, but it is certainly an important issue to be explored. Future research in this area can consider all these variables as well as including participants in the early stages of psychosis and individuals suffering other mental and/or organic illnesses.

Patient cultural background is probably an important variable affecting causal attribution and illness perception, although the present results neither corroborate nor refute this possibility. Ideas about the etiology of a mental disorder such as psychosis cannot be divorced from patient cultural context. For instance, in Latin cultures, family and society play influential roles in individual well-being [47] and are important etiological factors, although little research has been done on this phenomenon. To better identify any effect of culture, future research will need to replicate this study design in other Mexican populations living in the country or abroad.

Education of patients about the possible factors underlying psychosis onset and evolution and how treatment can positively influence its course is mandatory in clinical practice. Patients might well be aware of the biological aspects of their illness since these are most commonly emphasized by clinicians. However, the present study indicates that despite this education patients continue to believe that social and personality factors have the greatest effects on their condition. The patient attribution of illness to personality observed in the present results is probably associated with their perception of the negative effect it has on their life and emotional status. Exploration of patient beliefs about the etiology and effect of psychosis on their lives should be considered a regular part of clinical interviews. Acknowledging patient beliefs about their illness could lead to feelings of credibility and consequently help them to be more involved in and committed to treatment. Furthermore, eliciting the patients' point of view, in a certain way, can be considered a therapeutic goal in itself, allowing patients to play an active role into a highly desirable model of care, one that is based on partnership [48]. In addition, it could help clinicians to identify possible patient feelings of guilt, shame, or despair which might hinder recovery. Patients may identify social (i.e., external) factors as the primary cause of illness, but improving patients' confidence in terms of internal skills could support illness self-management. Considering patient beliefs about their illness could increase treatment adherence and decrease inadequate use of health services [49]. If the aim of intervention is an overall, qualitative recovery, treatment needs to go beyond mere symptom control and encompass patient perceptions of an illness's negative impact [15].

The present study is a preliminary contribution to better understanding of the beliefs of psychotic patients in Mexico about their illness. It can serve as a starting point for further research aimed at developing and implementing support programs and interventions for psychotic patients in Mexico, as well as Mexican (and alike) immigrants in other countries [40, 50, 51]. Knowledge of belief patterns in patients and the general population can help to design mental health awareness campaigns to increase community consciousness about mental illness, promote patient acceptance, and encourage the use of professional health services when needed.

## 5. Conclusions

Psychotic patients in a sample from Mexico were found to attribute onset of their illness mainly to social factors and to suggest that attribution of the illness to their personality could have a negative effect on their life. Promoting and maintaining awareness of patient beliefs and considering them in clinical practice could improve treatment efficacy and overall recovery. This is particularly vital in illnesses with severe and/or persistent symptoms, such as psychosis.

## Figures and Tables

**Table 1:** Causes of illness (Angermeyer and Klusmann Scale) as identified by patients (*N* = 62).

	(Very) likely	Scores (1–4)
	*n* (%)	Means (SDs)
Biology		10.4 (3.5)
Disturbance of brain biochemistry	32 (51.7)	2.47 (1.29)
Hereditary factors	24 (38.7)	2.23 (1.27)
Birth trauma	10 (16.2)	1.55 (0.95)
Brain injury	9 (14.6)	1.53 (0.94)
Organic disease external to brain	6 (9.7)	1.42 (0.76)
Infectious brain disease	4 (6.4)	1.19 (0.65)
Personality		11.9 (3.7)
Avoidance of everyday life problems	27 (43.6)	2.34 (1.16)
Failure in life	26 (41.9)	2.32 (1.18)
Lack of willpower	25 (40.4)	2.18 (1.18)
Too bright or intelligent	17 (27.4)	1.76 (1.10)
Too ambitious	14 (22.6)	1.71 (1.09)
Drug/alcohol abuse	12 (19.4)	1.60 (1.08)
Family		11.1 (4.5)
Broken home	21 (33.9)	2.00 (1.13)
Lack of parental love	19 (30.6)	2.03 (1.15)
Parent attitude hostile-rejecting	19 (30.6)	1.92 (1.16)
Father too severe	18 (29.0)	1.81 (1.13)
Overprotective mother	16 (25.8)	1.76 (1.14)
Parental expectations too high	9 (14.6)	1.53 (0.90)
Society		12.7 (4.1)
Stressful life events	35 (56.5)	2.60 (1.25)
Constant strain in school/job	29 (46.8)	2.35 (1.20)
Troubles in marriage/partnership	24 (38.7)	2.06 (1.28)
Society	23 (37.1)	2.02 (1.18)
Loneliness	20 (32.3)	2.00 (1.23)
Influence of bad friends	14 (22.6)	1.71 (1.06)
Esoteric		8.8 (3.1)
Possession by evil spirits	17 (27.5)	1.84 (1.22)
Lack of vitamins	14 (22.6)	1.77 (1.05)
Punishment by God	8 (12.9)	1.40 (0.91)
Unfavorable horoscope	6 (9.7)	1.29 (0.82)
Radiation	4 (6.4)	1.24 (0.62)
Environmental pollution	3 (4.8)	1.23 (0.53)

*Note.* Answers to each item were scored: (1) not a cause, (2) possible cause, (3) likely cause, and (4) very likely cause.

**Table 2:** Pearson correlations between illness perception dimensions (Brief-IPQ) and illness attribution categories (Angermeyer and Klusmann Scale) (*N* = 62).

	Biology	Personality	Family	Society	Esoteric
Cognitive	.14	.25^*^	.25^*^	.20	−.05
Emotional	.28^*^	.31^**^	.30^*^	.22	.04
Understanding	−.20	−.26^*^	−.25^*^	−.09	−.10

^*^ *P* ≤ .05; ^**^ *P* ≤ .01.

*Note.* Score range for the Brief-IPQ: from 1 to 4. High scores in the cognitive and emotional dimensions reflect an unfavorable perception of illness; high scores in the understanding dimension are favorable. Score range for the Angermeyer and Klusmann Scale: from 1 to 4. A high score reflects acknowledging that factor as a cause of illness.

**Table 3:** Significant correlations between illness perception items and illness attribution categories (*N* = 62).

Illness perception items	Illness attribution categories	*r*
(1) My illness affects my life^c^	Personality	.44^**^
	Family	.28^*^
	Society	.27^*^

(2) My illness will continue forever^c^	—	—

(3) I have extreme control over my illness^c^	—	—

(4) I am extremely concerned about my illness^e^	Biology	.29^*^
	Personality	.25^*^
	Family	.26^*^

(5) I very clearly understand my illness^u^	Personality	−.26^*^
	Family	−.25^*^

(6) My illness extremely affects me emotionally^e^	Personality	.30^*^
	Family	.26^*^
	Society	.27^*^

(7) Treatment is extremely helpful for my illness^c^	—	—

(8) I experience many severe symptoms from my illness^c^	—	—

^c^Cognitive dimension item, ^e^emotional dimension item, and ^u^understanding dimension item.

^*^ *P* ≤ .05, ^**^ *P* ≤ .001.

## References

[1] Corrigan P. W., Angell B., Davidson L., Marcus S. C., Salzer M. S., Kottsieper P., Larson J. E., Mahoney C. A., O'Connell M. J., Stanhope V. (2012). From adherence to self-determination: evolution of a treatment paradigm for people with serious mental illnesses. *Psychiatric Services*.

[2] Davidson L., Miller R., Flanagan E. (2008). What's in it for me? The utility of psychiatric treatments from the perspective of the person in recovery. *Epidemiologia e Psichiatria Sociale*.

[3] Topor A., Borg M., Mezzina R., Sells D., Marin I., Davidson L. (2006). Others: the role of family, friends, and professionals in the recovery process. *American Journal of Psychiatric Rehabilitation*.

[4] Vanheusden K., van der Ende J., Mulder C. L., Lenthe F. J., Verhulst F. C., Mackenbach J. P. (2009). Beliefs about mental health problems and help-seeking behavior in Dutch young adults. *Social Psychiatry and Psychiatric Epidemiology*.

[5] Khoury N. M., Kaiser B. N., Keys H. M., Brewster A.-R. T., Kohrt B. A. (2012). Explanatory models and mental health treatment: is vodou an obstacle to psychiatric treatment in rural Haiti?. *Culture, Medicine and Psychiatry*.

[6] Petrie K. J., Jago L. A., Devcich D. A. (2007). The role of illness perceptions in patients with medical conditions. *Current Opinion in Psychiatry*.

[7] Shah P., Hull T., Riley G. A. (2009). Associations between the illness perception questionnaire for schizophrenia and engagement in treatment in a secure setting. *Clinical Psychologist*.

[8] Williams K., Steer H. (2011). Illness perceptions: are beliefs about mental health problems associated with self-perceptions of engagement in people with psychosis?. *Behavioural and Cognitive Psychotherapy*.

[9] Botha-Scheepers S., Riyazi N., Kroon H. M., Scharloo M., Houwing-Duistermaat J. J., Slagboom E., Rosendaal F. R., Breedveld F. C., Kloppenburg M. (2006). Activity limitations in the lower extremities in patients with osteoarthritis: the modifying effects of illness perceptions and mental health. *Osteoarthritis and Cartilage*.

[10] Scharloo M., Kaptein A. A., Weinman J., Bergman W., Vermeer B. J., Rooijmans H. G. M. (2000). Patients' illness perceptions and coping as predictors of functional status in psoriasis: a 1-year follow-up. *British Journal of Dermatology*.

[11] Frostholm L., Fink P., Christensen K. S., Toft T., Oernboel E., Olesen F., Weinman J. (2005). The patients' illness perceptions and the use of primary health care. *Psychosomatic Medicine*.

[12] Levinson C. M., Druss B. G. (2005). Health beliefs and depression in a group of elderly high utilizers of medical services. *General Hospital Psychiatry*.

[13] Lobban F., Barrowclough C., Jones S. (2004). The impact of beliefs about mental health problems and coping on outcome in schizophrenia. *Psychological Medicine*.

[14] Scharloo M., Kaptein A. A., Schlösser M., Pouwels H., Bel E. H., Rabe K. F., Wouters E. F. M. (2007). Illness perceptions and quality of life in patients with chronic obstructive pulmonary disease. *Journal of Asthma*.

[15] Stainsby M., Sapochnik M., Bledin K., Mason O. J. (2010). Are attitudes and beliefs about symptoms more important than symptom severity in recovery from psychosis?. *Psychosis*.

[16] Theodore K., Johnson S., Chalmers-Brown A., Doherty R., Harrop C., Ellett L. (2012). Quality of life and illness beliefs in individuals with early psychosis. *Social Psychiatry and Psychiatric Epidemiology*.

[17] Watson P. W. B., Garety P. A., Weinman J., Dunn G., Bebbington P. E., Fowler D., Freeman D., Kuipers E. (2006). Emotional dysfunction in schizophrenia spectrum psychosis: the role of illness perceptions. *Psychological Medicine*.

[18] Mendenhall E., Fernandez A., Adler N., Jacobs E. A. (2012). Susto, coraje, and abuse: depression and beliefs about diabetes. *Culture, Medicine, and Psychiatry*.

[19] Leventhal H., Nerenz D. R., Steele D. J., Baum A., Taylor S. E., Singer J. E. (1984). Illness representations and coping with health threats. *Handbook of Psychology and Health: Social Psychological Aspects of Health*.

[20] Lau R. R., Bernard T. M., Hartman K. A. (1989). Further explorations of common-sense representations of common illnesses. *Health Psychology*.

[21] Broadbent E., Petrie K. J., Main J., Weinman J. (2006). The brief illness perception questionnaire. *Journal of Psychosomatic Research*.

[22] Holzinger A., Müller P., Priebe S., Angermeyer M. C. (2001). Etiology of schizophrenia from the viewpoint of the patient. *Psychiatrische Praxis*.

[23] Angermeyer M. C., Klusmann D. (1988). The causes of functional psychoses as seen by patients and their relatives. I. The patients' point of view. *European Archives of Psychiatry and Neurological Sciences*.

[24] Angermeyer M. C., Klusmann D., Walpuski O. (1988). The causes of functional psychoses as seen by patients and their relatives. II. The relatives' point of view. *European Archives of Psychiatry and Neurological Sciences*.

[25] Esterberg M. L., Compton M. T. (2006). Causes of schizophrenia reported by family members of urban African American hospitalized patients with schizophrenia. *Comprehensive Psychiatry*.

[26] Holzinger A., Müller P., Priebe S., Angermeyer M. C. (2001). Etiology of schizophrenia from the viewpoint of family. *Psychiatrische Praxis*.

[27] Petrie K. J., Weinman J. (2006). Why illness perceptions matter. *Clinical Medicine*.

[28] World Medical Association (2008). *World Medical Association Declaration of Helsinki: Ethical Principles for Medical Research Involving Human Subjects*.

[29] Gobierno Constitucional de los Estados Unidos Mexicanos Diario Oficial de la Federación. Reglamento de la Ley General de Salud en Materia de Investigación para la Salud. http://www.ordenjuridico.gob.mx/leyes.php.

[30] American Psychiatric Association (2000). *Diagnostic and Statistical Manual of Mental Disorders*.

[31] Pacheco-Huergo V., Viladrich C., Pujol-Ribera E., Cabezas-Peña C., Núñez M., Roura-Olmeda P., Amado-Guirado E., Núñez E., Del Val J. L. (2012). Perception in chronic illnesses: linguistic validation of the revised Illness Perception Questionnaire and the Brief Illness Perception Questionnaire for a Spanish population. *Atención Primaria*.

[32] Broadbent E., Kydd R., Sanders D., Vanderpyl J. (2008). Unmet needs and treatment seeking in high users of mental health services: role of illness perceptions. *Australian and New Zealand Journal of Psychiatry*.

[33] Bazzazian S., Besharat M. A. (2010). Reliability and validity of a Farsi version of the brief illness perception questionnaire. *Procedia—Social and Behavioral Sciences*.

[34] de Raaij E. J., Schröder C., Maissan F. J., Pool J. J., Wittink H. (2012). Cross-cultural adaptation and measurement properties of the brief illness perception questionnaire-Dutch language version. *Manual Therapy*.

[35] Hallegraeff J. M., van der Schans C. P., Krijnen W. P., de Greef M. H. G. (2013). Measurement of acute nonspecific low back pain perception in primary care physical therapy: reliability and validity of the brief illness perception questionnaire. *BMC Musculoskeletal Disorders*.

[36] Goulding S. M., Broussard B., Demir B., Compton M. T. (2009). An exploration of the factor structure and development of potentially useful subscales of etiological beliefs about schizophrenia in a general population sample. *Social Psychiatry and Psychiatric Epidemiology*.

[37] Baer R. D., Weller S. C., de Alba Garcia J. G., Glazer M., Trotter R., Pachter L., Klein R. E. (2003). A cross-cultural approach to the study of the folk illness nervios. *Culture, Medicine and Psychiatry*.

[38] Cartwright E. (2007). Bodily remembering: memory, place, and understanding Latino folk illnesses among the Amuzgos Indians of Oaxaca, Mexico. *Culture, Medicine, and Psychiatry*.

[39] Salgado de Snyder V. N., Diaz-Perez M. D. J., Ojeda V. D. (2000). The prevalence of nervios and associated symptomatology among inhabitants of Mexican rural communities. *Culture, Medicine and Psychiatry*.

[40] Lewis-Fernández R., Horvitz-Lennon M., Blanco C., Guarnaccia P. J., Cao Z., Alegría M. (2009). Significance of endorsement of psychotic symptoms by US latinos. *The Journal of Nervous and Mental Disease*.

[41] Molvaer J., Hantzi A., Papadatos Y. (1992). Psychotic patients' attributions for mental illness. *British Journal of Clinical Psychology*.

[42] World Health Organization Social determinants of health. http://www.who.int/social_determinants/en/.

[43] Harrow M., Hansford B. G., Astrachan-Fletcher E. B. (2009). Locus of control: Relation to schizophrenia, to recovery, and to depression and psychosis—a 15-year longitudinal study. *Psychiatry Research*.

[44] Wu A. M. S., Tang C. S.-K., Kwok T. C. Y. (2004). Self-efficacy, health locus of control, and psychological distress in elderly Chinese women with chronic illnesses. *Aging and Mental Health*.

[45] Schmutte T., Flanagan E., Bedregal L., Ridgway P., Sells D., Styron T., Davidson L. (2009). Self-efficacy and self-care: missing ingredients in health and healthcare among adults with serious mental illnesses. *The Psychiatric Quarterly*.

[46] Apiquián R., Páez F., Loyzaga C. (1997). Estudio Mexicano sobre el primer episodio psicótico: resultados preliminares, características sociodemográficas y clínicas. *Salud Mental*.

[47] Caqueo-Urízar A., Lemos-Giráldez S. (2008). Calidad de vida y funcionamiento familiar de pacientes con esquizofrenia en una comunidad Latinoamericana. *Psicothema*.

[48] Velpry L. (2008). The patient's view: issues of theory and practice. *Culture, Medicine and Psychiatry*.

[49] Petrie K. J., Broadbent E., Kydd R. (2008). Illness perceptions in mental health: issues and potential applications. *Journal of Mental Health*.

[50] Andrés-Hyman R. C., Ortiz J., Añez L. M., Paris M., Davidson L. (2006). Culture and clinical practice: recommendations for working with Puerto Ricans and other Latinas(os) in the United States. *Professional Psychology: Research and Practice*.

[51] Añez L. M., Paris M., Bedregal L. E., Davidson L., Grilo C. M. (2005). Application of cultural constructs in the care of first generation Latino clients in a community mental health setting. *Journal of Psychiatric Practice*.

